# Pilot randomized controlled trial of restricted versus liberal crystalloid fluid management in pediatric post-operative and trauma patients

**DOI:** 10.1186/s40814-023-01408-w

**Published:** 2023-11-08

**Authors:** Vincent P. Duron, Rika Ichinose, Latoya A. Stewart, Chloe Porigow, Weijia Fan, Jeanne M. Rubsam, Steven Stylianos, Nicolino V. Dorrello

**Affiliations:** 1 Division of Pediatric Surgery, Morgan Stanley Children’s Hospital/New York-Presbyterian, Columbia University College of Physicians & Surgeons, , 3959 Broadway, CHN 215, New York, NY 10032 USA; 2 Columbia University Vagelos College of Physicians and Surgeons, 630W 168Th Street, New York, NY 10032 USA; 3 Department of Biostatistics, Columbia University Mailman School of Public Health, 722W 168Th Street, New York, NY 10032 USA; 4 Department of Pediatric Critical Care, CUIMC/New York-Presbyterian Morgan Stanley Children’s Hospital, New York City, USA

**Keywords:** Pediatric trauma, Fluid management, Fluid resuscitation, Feasibility, Randomized controlled trial

## Abstract

**Background:**

Intravenous (IV) fluid therapy is essential in the treatment of critically ill pediatric surgery and trauma patients. Recent studies have suggested that aggressive fluids may be detrimental to patients. Prospective studies are needed to compare liberal to restricted fluid management in these patients. The primary objective of this pilot trial is to test study feasibility—recruitment and adherence to the study treatment algorithm.

**Methods:**

We conducted a two-part pilot randomized controlled trial (RCT) comparing liberal to restricted crystalloid fluid management in 50 pediatric post-operative (1–18 years) and trauma (1–15 years) patients admitted to our pediatric intensive care unit (PICU). Patients were randomized to a high (liberal) volume or low (restricted) volume algorithm using unblinded, blocked randomization. A revised treatment algorithm was used after the 29th patient for the second part of the RCT. The goal of the trial was to determine the feasibility of conducting an RCT at a single site for recruitment and retention. We also collected data on the safety of study interventions and clinical outcomes, including pulmonary, infectious, renal, post-operative, and length of stay outcomes.

**Results:**

Fifty patients were randomized to either liberal (*n* = 26) or restricted (*n* = 24) fluid management strategy. After data was obtained on 29 patients, a first study analysis was performed. The volume of fluid administered and triggers for intervention were adapted to optimize the treatment effect and clarity of outcomes. Updated and refined fluid management algorithms were created. These were used for the second part of the RCT on patients 30–50. During this second study period, 54% (21/39, 95% CI 37–70%) of patients approached were enrolled in the study. Of the patients enrolled, 71% (15/21, 95% CI 48–89%) completed the study. This met our a priori recruitment and retention criteria for success. A data safety monitoring committee concluded that no adverse events were related to study interventions. Although the study was not powered to detect differences in outcomes, after the algorithm was revised, we observed a non-significant trend towards improved pulmonary outcomes in patients on the restricted arm, including decreased need for and time on oxygen support and decreased need for mechanical ventilation.

**Conclusion:**

We demonstrated the feasibility and safety of conducting a single-site RCT comparing liberal to restricted crystalloid fluid management in critically ill pediatric post-operative and trauma patients. We observed trends in improved pulmonary outcomes in patients undergoing restricted fluid management. A definitive multicenter RCT comparing fluid management strategies in these patients is warranted.

**Trial registration:**

ClinicalTrials.gov, NCT04201704. Registered 17 December 2019—retrospectively registered.

**Supplementary Information:**

The online version contains supplementary material available at 10.1186/s40814-023-01408-w.

## Key messages regarding feasibility

- We aim to perform a multicenter randomized controlled trial comparing liberal to restricted crystalloid fluid management in critically ill pediatric post-operative and trauma patients. It is unknown if participants and their families will agree to enroll in the study if the treatment algorithm will be adhered to, if the outcome measures will be collected, and if the study is safe.
- This trial found that patient enrollment and adherence to study procedures were feasible. Data collection was completed on all patients and the study was found to be safe with no study-related adverse events.
- We demonstrated the feasibility and safety of conducting the RCT at our institution and plan to use the results of the study to design a multicenter RCT to determine optimal fluid management strategies in this patient population.

## Background

Trauma is the number one cause of mortality in children over the age of 1 year [1, 2]. Intravenous fluid (IV) administration is a cornerstone of fluid management of post-operative and trauma patients. Approximately 250,000 children are admitted to the pediatric intensive care unit (PICU) each year in the USA [3]. Among them, approximately 35% are admitted after surgery or trauma for which IV fluid therapy is a key component of their management [4, 5]. IV fluid replacement restores intravascular volume, increases cardiac output, and improves end-organ oxygen delivery. Critically ill post-operative and trauma patients experience a severe inflammatory response that causes a capillary leak syndrome in which fluid extravasates out of capillaries into the surrounding interstitial space, thus requiring fluid resuscitation to maintain intravascular volume and hemodynamic stability [6]. Yet, despite significant advances in other aspects of surgical and trauma care, little emphasis has been placed on describing the optimal IV fluid management strategy in children and no fluid management guidelines have been elaborated to direct fluid treatment in these patients.

Recent randomized control trials (RCTs) in adult medical, surgical, and trauma patients have demonstrated that administration of high volumes of crystalloid may be associated with adverse clinical outcomes, including increased mortality, days on the ventilator, and ICU and hospital length of stay [7–9]. Studies in surgical, neurosurgical, burn, and trauma patients have demonstrated that restricting fluids may decrease the incidence of death and complications including cardiopulmonary complications, acute respiratory distress syndrome (ARDS), multi-organ failure, abdominal compartment syndrome, surgical site infections, bloodstream infections, and gastrointestinal complications [10–15]. Although retrospective studies have shown similar results in critically ill children, data is lacking in pediatric surgical and trauma patients, who may have a different physiologic response to stress than their adult counterparts [16, 17].

### Objectives

High-quality prospective studies are needed in pediatric post-operative and trauma patients to identify the optimal fluid strategy and timing of intervention in these patients. The primary aim of our pilot study was to demonstrate feasibility, safety, and eventually a treatment effect of a single institution randomized controlled trial comparing a liberal to a restrictive fluid management strategy in pediatric critically ill post-operative and trauma patients.

A second objective of the study was to devise a fluid treatment algorithm that may be used in PICUs in a variety of resource settings where complex instruments or ultra-specialized skillsets might not be available. For example, transthoracic echocardiography has been shown to provide reliable volume status assessment in real-time [18]. However, this specialized skillset is not available in all PICUs. Central venous monitoring has also been shown to correlate with volume status; however, it is often not reliable in children, particularly small children [19–22]. Furthermore, central venous catheters are not regularly placed in pediatric trauma and post-operative patients.

## Methods

### Study design

This study was composed of two consecutive prospective, single institution, un-blinded, randomized pilot studies on 29 and 21 (50 total) patients to determine the feasibility of conducting an RCT at our institution, acceptance by pediatric trauma and Pediatric Intensive Care Unit (PICU) teams, adherence to the protocol, treatment effect, and safety of the intervention. After analysis of the first 29 patients included in the first phase of this study, algorithm revisions were made before initiating the second phase of the study, which was analyzed separately due to treatment protocol changes. The study was approved by the Columbia University Irving Medical Center Institutional Review Board (CUIMC IRB) Committee (n. AAAR2083) and registered under the ClinicalTrials.gov Identifier NCT04201704.

**Fig. 1:**
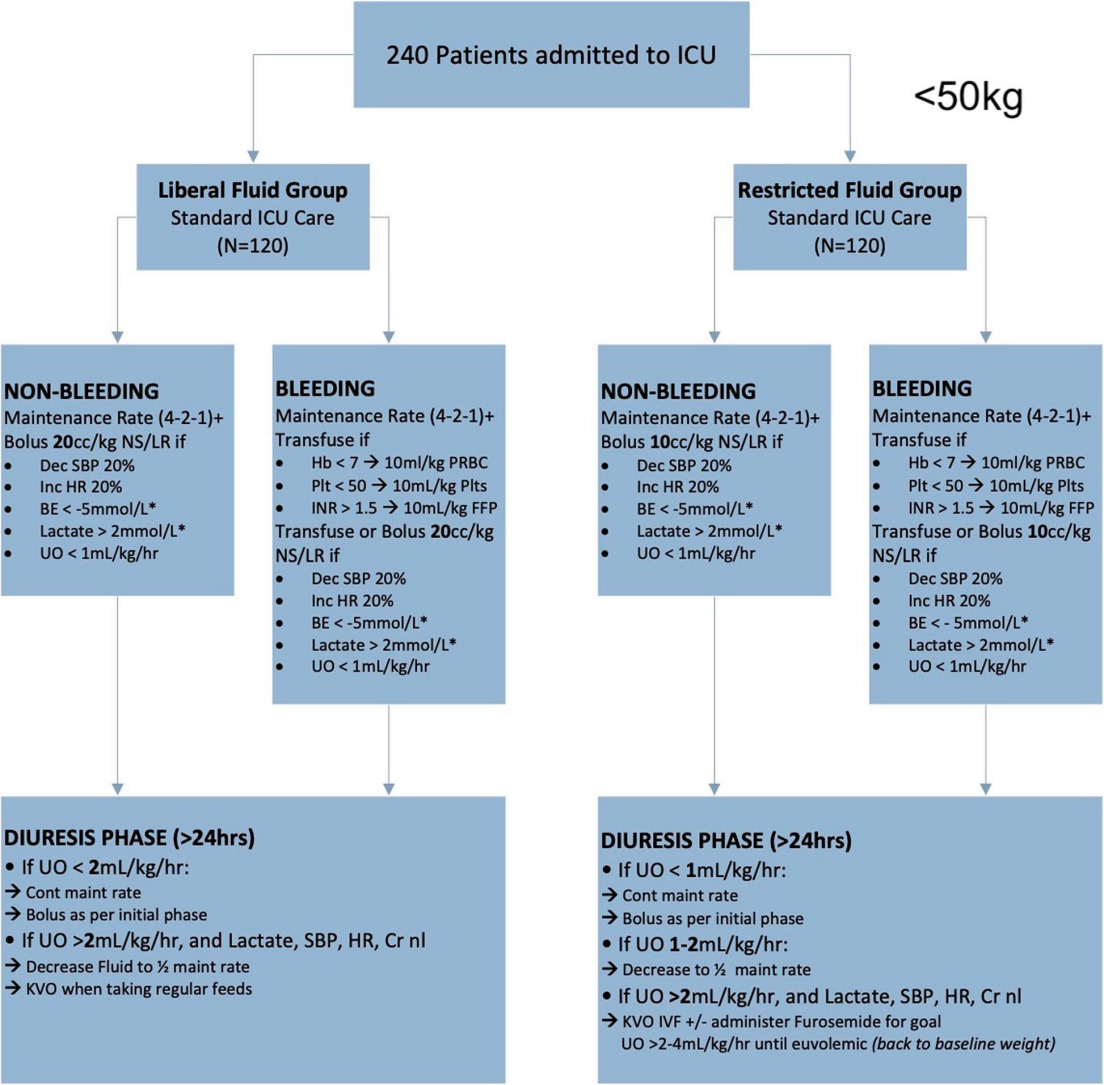
Initial treatment algorithm for patients < 50 kg

**Fig. 2:**
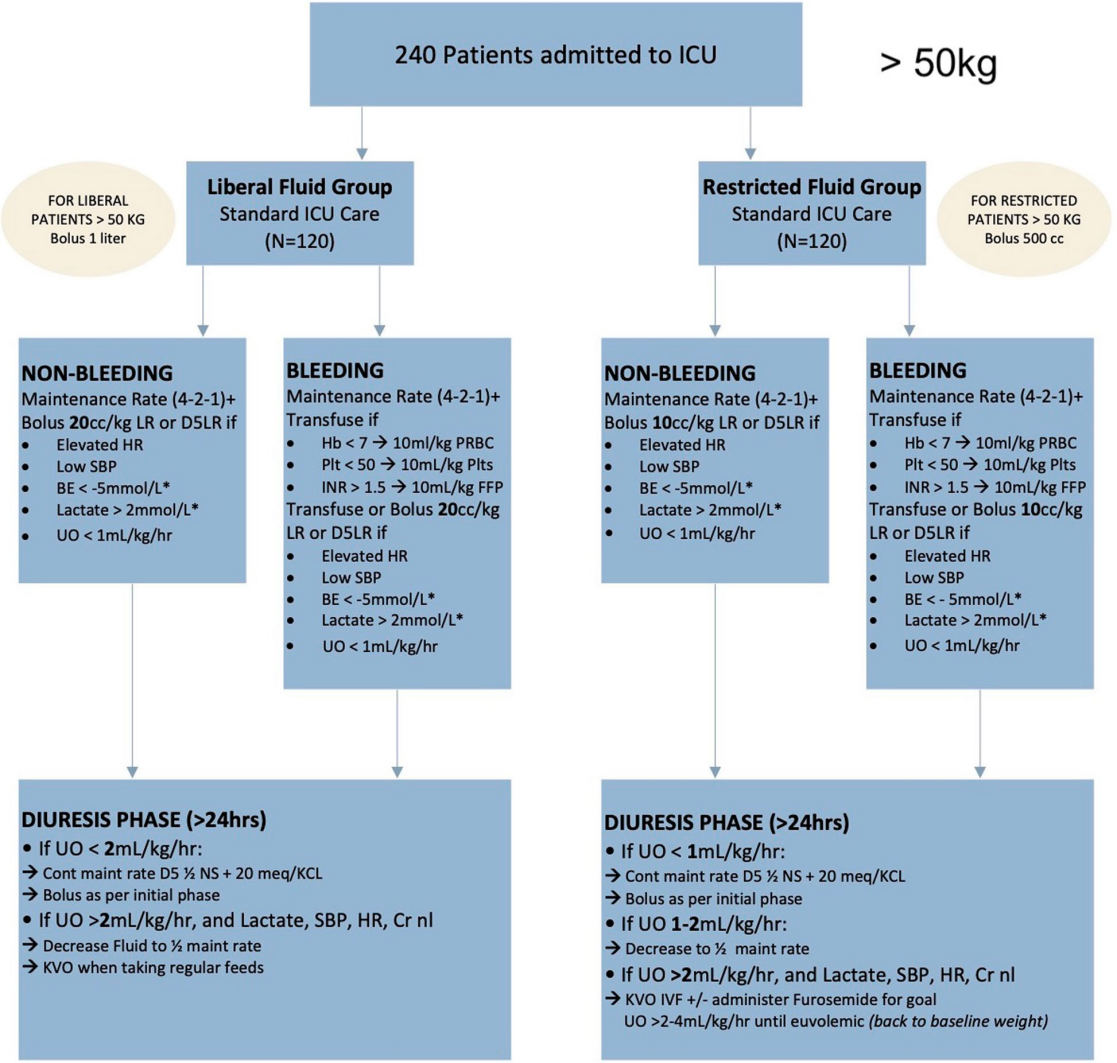
Initial treatment algorithm for patients > 50 kg

Focus groups between co-investigators and advisors from the PICU and trauma services were conducted to establish the goals of the study, the inclusion and exclusion criteria, the variables measured, and the intervention triggers used to guide fluid therapy in the study patients. A study algorithm for a liberal and restricted arm based on multiple adult fluid algorithms was tailored specifically to pediatric patients. Physiologic parameters that could most accurately reflect intravascular volume were identified and incorporated into the algorithms to guide fluid management. The algorithm was divided into two fluid therapy phases. The “resuscitation” phase was defined as the phase of a capillary leak during which patients require crystalloid fluid therapy to maintain intravascular volume. The second phase of therapy, the “diuresis” phase, starts after patients have been resuscitated and regained euvolemia, no longer require crystalloid fluid therapy to replete intravascular volume and may benefit from diuretic therapy. The algorithm was extensively reviewed and vetted during the focus groups and agreed upon. Physiologic parameters that could most accurately reflect intravascular volume were identified and incorporated into the algorithms to guide fluid management (Fig. 1 depicts the algorithm for patients < 50 kg; Fig. 2 depicts the algorithm for patients > 50 kg).

**Fig. 3:**
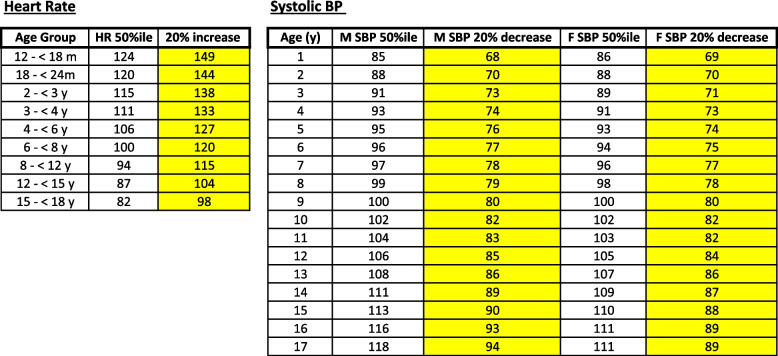
Algorithm reference tables for HR and SBP

In our pilot study, we ensured that no additional blood draws or interventions would be performed on study patients. Thus, the laboratory values that are incorporated into the algorithm are not required elements, but rather variables that—if drawn for non-study reasons—may support the decision-making process in the assessment of patient volume status. Specifically, systolic blood pressure (SBP), heart rate (HR), base excess (BE), serum lactate levels, and urine output were used to guide our fluid therapy. Algorithmic reference normal values for HR and SBP were based on a literature review and are displayed in Fig. 3. HR increase of 20% and SPB decrease of 20% from the referenced 50th percentiles were considered criteria for intervention [23, 24]. The maintenance fluid rate starts in the PICU, at the time the patients are enrolled into the study. Their fluid maintenance rate is calculated as specified in the protocol. Fluids administered prior to starting the study protocol are not considered in the total ins and outs of crystalloid fluid administered. For the first study, patients were separated into bleeding and non-bleeding groups. These labels were assigned to the patient by the operating surgeon or trauma surgeon at the beginning of the study. For example, a patient that is expected to require blood transfusions will be labeled as “bleeding”. For bleeding patients, once they were no longer requiring blood product administration, they were automatically transitioned to the non-bleeding arm, which followed the same criteria for crystalloid management as the bleeding arm.

### Outcomes

The primary outcome was overall complications up to discharge, a binary indicator for whether the subject has had any complications after being admitted to the PICU. These complications included: major complications—pulmonary edema, hemorrhage, hardware/deep cavity infection, anastomotic dehiscence, thrombosis, acute respiratory distress syndrome, abdominal compartment syndrome, or death; and minor complications—superficial wound infection, ileus, pneumonia. We estimated and compared the proportion of complications between the two groups using Pearson’s chi-squared test.

Secondary outcomes included the number of days on supplemental oxygen, the number of days on ventilator, ICU length of stay, overall hospital length of stay, and each of the individual complications. For binary secondary outcomes, we compared proportions and performed Pearson’s chi-squared tests. For quantitative outcomes, we examined the distribution of the variables and performed analyses as appropriate. For example, for quantitative variables that were normal or close to normal, we used 2 sample *t* tests. On the other hand, for quantitative variables that were very non-normal, we used nonparametric methods.

The outcomes of this feasibility study were analyzed separately for the two phases of this study. The pilot study was separated into two analyses so that adjustments could be made to the algorithms based on the results after the first analysis [18–22, 25].

### Participants

Both critically ill post-operative and trauma patients were included as they have a similar systemic inflammatory response described above and thus the same physiologic requirements and response to crystalloids. The exclusion and inclusion criteria are listed in Table 1.

**Table 1:** Inclusion and exclusion criteria

Inclusion criteria	Exclusion criteria
• Trauma patients 1 year old to 15 years old admitted to the PICU • Post-operative patients 1 year old to 18 years old admitted to the PICU–general surgery, urology, orthopedic patients • Patients admitted to the PICU directly from the ED • Patients admitted to the PICU from the OR • Patients transferred to PICU from outside facility ER (need to have been in ER 12 h or less)	• Patients transferred to PICU from outside PICU or floor • Patients transferred to PICU from outside ER if > 12 h • Patient with congenital heart disease as defined by a congenital cardiac defect requiring surgery or medication • Patient with a diagnosis of a chronic cardiac condition (e.g. hypertension, cardiac arrhythmia) • Patients with chronic kidney disease as defined by an abnormality of kidney stricture or function, present for more than 3 months, with implications for health • Post-operative transplant, cardiac, and neurosurgical patients • Patients with traumatic brain injury • Patients with any disease that may affect baseline blood pressure and heart rate (endocrine disorders, certain genetic disorders, mitochondrial diseases) • Hypotension requiring vasopressor therapy • If a massive transfusion protocol initiated

### Study procedures and randomization

The first contact with a potential subject was made by the treating physician when possible. Recruitment occurred in the ED or the PICU. Study participants were required to consent and enroll into the study before 12 h had elapsed from the time they arrived in the PICU. This time frame was chosen to allow families to thoughtfully and freely decide to participate in the study, while also limiting the number of non-study interventions that take place prior to the start of study interventions.

After obtaining informed consent, research staff created a patient record, and patients were randomized through Research Electronic Data Capture (REDCap) to be enrolled into the restricted or liberal fluid arm. Initial in-servicing of PICU attendings, housestaff, and nursing staff was undertaken prior to the onset of the study in the form of PowerPoint presentations that reviewed the algorithm, inclusion and exclusion criteria, and study interventions. For each patient enrolled, the study PI or designated research team member engaged with the PICU in person to discuss the patient with the treating team—physician, nurse practitioner, resident, and bedside nurse. A laminated sheet detailing the specific arm of the study the patient was enrolled in was left with the bedside nurse. Direct contact with the study team was available 24 h a day. The data was entered into REDCap daily.

### Feasibility of recruitment

Recruitment for both study periods was conducted between August 2018 to January 2021. An enrollment log was consistently recorded for the second study period while recruiting study participants 30–50. A total of 39 patients were approached for eligibility; 16 declined to participate; 2 consented but were excluded because they did not end up being admitted to the PICU. Of the 21 patients enrolled, 12 were randomized to “liberal” and 9 were randomized to “restricted” fluid management. Six patients did not complete the study due to off-protocol treatment with extra fluid bolus or administration of pressors, and in one case, the parent withdrew consent. Thus, during the second study period, 15 of 21 (71%) of enrolled patients successfully completed the study. The pilot study was stopped when 50 patients were recruited and enrolled. Recruitment was most effective when the PI or the nurse practitioner on the study team approached the parent or guardian for consent.

### Data safety

A Data Safety Monitoring Committee (DSMC) was established in accordance with National Institutes of Health (NIH) requirements for multisite clinical trials. By definition, DSMC is entirely independent of any trial investigator and any site that may be actively enrolling patients in the study. The DSMC met after 25 patients were recruited and again after 50 patients. Data was analyzed in two separate analyses. Co-chairs for the DSMC were designated according to their clinical expertise, research experience and independence from the study. Adverse Events (AEs) include complications that are primary and secondary outcomes of this trial. All serious, unexpected, or unanticipated, and possibly related to the study treatments AEs were to be reported within 48 h to the IRB. They were classified by Systems Organ Class and Preferred Term [26]. All AEs begin after randomization and through the period of hospitalization. AEs are coded using the latest version of the Medical Dictionary for Regulatory Activities.

### Statistical analysis

Blocked randomization list was created with permuted block sizes of 2 and 4 to randomly assign patients to either the liberal fluid arm or the restricted fluid arm. REDCap is used to collect and store data.. REDCap is a secure, web-based application designed to support data capture for research studies, providing: (1) an intuitive interface for validated data entry; (2) audit trails for tracking data manipulation and export procedures; (3) automated export procedures for seamless data downloads to common statistical packages that will be used by the statistician; and (4) procedures for importing data from external sources.

The data was analyzed using intent-to-treat analyses. Upon obtaining the data, we evaluated the comparability of key demographic variables and relevant baseline clinical variables between the two treatment arms. A complete list of the variables that were recorded in REDCap and analyzed are listed in the supplemental materials.

Frequency with proportion was reported for categorical variables while median with interquartile range (IQR) was reported for continuous variables. We estimated and compared the proportion of categorical variables between the two groups using Fisher’s exact test. For continuous variables, the Wilcoxon rank sum test was used. Data was analyzed in two separate analyses for the two phases of the study. Analysis was conducted in R version 4.2.0.

## Results

### Sample

**Fig. 4:**
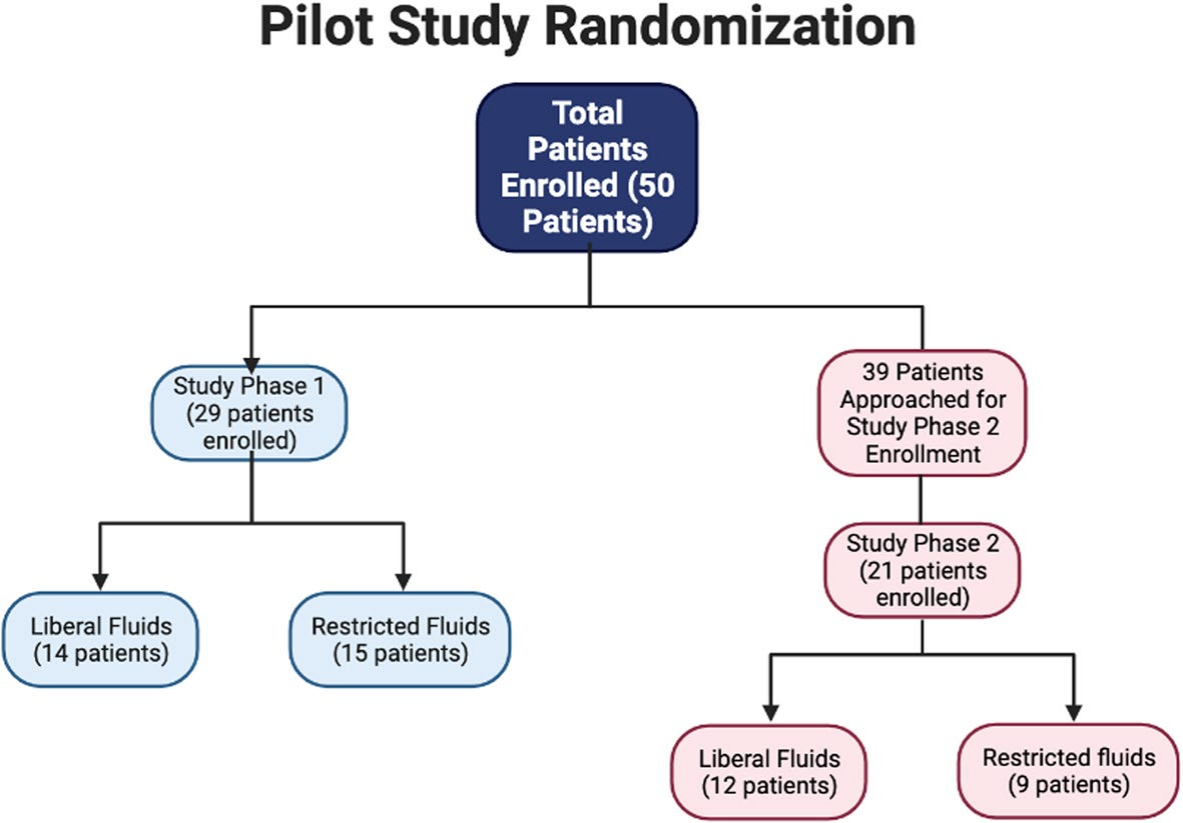
Participant randomization flow diagram

Fifty (50) critically ill pediatric post-operative or trauma patients admitted to our PICU were enrolled in the study, according to the inclusion and exclusion criteria described previously (Table 1). Written informed consent from the parents and approval from the attending physicians were obtained. Assent was obtained from patients when they were more than 7 years old. Baseline demographic data of both study arms was collected (Table 2). The number of trauma patients and number of post-operative patients recruited from each specialty was similar (Fig. 4). Trauma patients had Injury Severity (ISS) scores of 9, 10, 10, 14, and 14, with mechanisms of injury including gunshot wound (GSW), motor vehicle collision (MVC), and fall. Some trauma patients required surgery. Most surgical patients were general surgery or orthopedic surgery patients. General surgery procedures included congenital diaphragmatic hernia repair, choledochal cyst excision, laparotomy, ileocectomy, ventral hernia repair, colostomy or ileostomy takedown or creation, thymectomy, ileoanal pouch creation, and abdominal wall reconstruction. Some patients had surgery from more than one service.

**Table 2:** Baseline demographic characteristics and admission vitals (subjects 1–29 and 30–50)

Subjects 1–29 patient characteristics	Liberal (*n* = 14)	Restricted (*n* = 15)	Total (*n* = 29)
Female sex	8 (57.1%)	8 (53.3%)	16 (55.2%)
Non-bleeding arm	9 (64.3%)	13 (86.7%)	22 (75.9%)
Age, median (IQR)	10.300 (6.025, 12.775)	13.800 (7.850, 14.500)	11.900 (7.600, 14.100)
Weight in kg, median (IQR)	31.100 (22.425, 42.850)	33.000 (25.650, 52.550)	32.200 (23.700, 46.400)
Height in cm, median (IQR)	137.000 (115.500, 150.250)	132.000 (123.000, 155.000)	135.000 (120.000, 153.000)
Trauma case	3 (21.4%)	0 (0.0%)	3 (10.3%)
Comorbidity	11 (78.6%)	14 (93.3%)	25 (86.2%)
Peds surgery service	5 (35.7%)	5 (33.3%)	10 (34.5%)
Peds urology service	1 (7.1%)	1 (6.7%)	2 (6.9%)
Peds ortho service	7 (50.0%)	10 (66.7%)	17 (58.6%)
Other peds service	1 (7.1%)	1 (6.7%)	2 (6.9%)
Total crystalloid in cc/kg/hr, median (IQR)	2.915 (2.119, 4.148)	2.458 (1.643, 3.612)	2.659 (1.952, 3.804)
Total time on protocol in hrs, median (IQR)	26.950 (12.492, 40.979)	27.833 (15.525, 41.292)	27.000 (11.950, 41.317)
Admission pH, median (IQR)	7.390 (7.310, 7.410)	7.370 (7.330, 7.410)	7.380 (7.312, 7.410)
Admission HCO3, median (IQR)	24.150 (23.350, 25.250)	23.650 (23.025, 24.425)	23.900 (23.200, 24.950)
Admission Lactate, median (IQR)	1.150 (0.825, 1.675)	1.000 (0.700, 1.925)	1.100 (0.775, 1.875)
Admission sodium, median (IQR)	140.000 (140.000, 142.000)	141.500 (140.000, 142.750)	141.000 (140.000, 142.500)
Admission hemoglobin, median (IQR)	9.300 (8.925, 10.200)	10.750 (10.125, 11.250)	10.200 (8.975, 11.025)
Admission creatinine, median (IQR)	0.400 (0.200, 0.420)	0.405 (0.370, 0.595)	0.400 (0.290, 0.545)
Admission white blood cell count, median (IQR)	11.325 (8.575, 14.900)	12.790 (9.117, 17.095)	11.680 (8.723, 15.873)
Admission platelet count, median (IQR)	225.000 (184.250, 267.750)	220.000 (160.250, 293.750)	220.000 (172.250, 279.000)
Admission BUN, median (IQR)	6.000 (5.000, 10.000)	8.500 (8.000, 10.750)	8.000 (6.000, 10.500)
Admission INR, median (IQR)	1.500 (1.400, 1.600)	1.600 (1.350, 1.700)	1.550 (1.375, 1.650)
Admission APTT, median (IQR)	32.600 (30.300, 32.900)	27.350 (25.175, 29.525)	31.700 (28.800, 32.750)
Subjects 30–50 patient characteristics	Liberal (*n* = 12)	Restricted (*n* = 9)	Total (*n* = 21)
Female sex	8 (66.7%)	5 (55.6%)	13 (61.9%)
Non-bleeding arm	10 (83.3%)	8 (88.9%)	18 (85.7%)
Age, median (IQR)	11.700 (10.150, 13.525)	9.900 (5.700, 11.900)	11.500 (8.500, 12.800)
Weight in kg, median (IQR)	35.450 (24.900, 49.175)	28.000 (17.700, 36.700)	28.000 (21.000, 44.700)
Height in cm, median (IQR)	128.000 (100.000, 145.500)	116.000 (109.000, 140.000)	126.000 (100.000, 140.000)
Trauma case	0 (0.0%)	2 (22.2%)	2 (9.5%)
Comorbidity	11 (91.7%)	5 (55.6%)	16 (76.2%)
Peds surgery service	4 (33.3%)	2 (22.2%)	6 (28.6%)
Peds urology service	0 (0%)	0 (0%)	0 (0%)
Peds ortho service	10 (83.3%)	6 (66.7%)	16 (76.2%)
Other peds service	0 (0%)	0 (0%)	0 (0%)
Total crystalloid in cc/kg/h, median (IQR)	2.567 (1.527, 3.095)	1.602 (1.523, 2.434)	2.336 (1.506, 2.893)
Total time on protocol in hrs, median (IQR)	20.183 (12.750, 26.708)	12.000 (6.858, 35.308)	19.600 (7.833, 29.325)
Admission pH, median (IQR)	7.370 (7.330, 7.385)	7.360 (7.320, 7.385)	7.370 (7.330, 7.390)
Admission HCO3, median (IQR)	25.200 (24.150, 26.300)	21.750 (21.075, 25.350)	24.900 (22.200, 26.400)
Admission Lactate, median (IQR)	0.800 (0.800, 1.650)	1.150 (0.650, 1.725)	1.000 (0.800, 1.900)
Admission sodium, median (IQR)	139.500 (139.000, 140.250)	139.000 (136.000, 141.000)	139.000 (138.000, 141.000)
Admission hemoglobin, median (IQR)	10.650 (9.700, 11.850)	10.900 (10.400, 11.500)	10.800 (10.025, 11.550)
Admission creatinine, median (IQR)	0.550 (0.335, 0.610)	0.450 (0.340, 0.600)	0.480 (0.340, 0.600)
Admission white blood cell count, median (IQR)	15.120 (12.915, 17.593)	15.100 (10.763, 23.598)	15.120 (12.578, 20.058)
Admission platelet count, median (IQR)	212.000 (169.250, 242.500)	220.500 (185.500, 254.000)	217.000 (176.250, 250.750)
Admission BUN, median (IQR)	8.000 (7.000, 10.000)	9.000 (7.000, 11.000)	8.000 (7.000, 11.000)
Admission INR, median (IQR)	1.300 (1.300, 1.675)	1.200 (1.200, 1.300)	1.300 (1.200, 1.400)
Admission APTT, median (IQR)	29.800 (29.000, 35.800)	30.550 (29.525, 31.575)	29.800 (28.950, 34.200)

### First study analysis outcomes

**Fig. 5:**
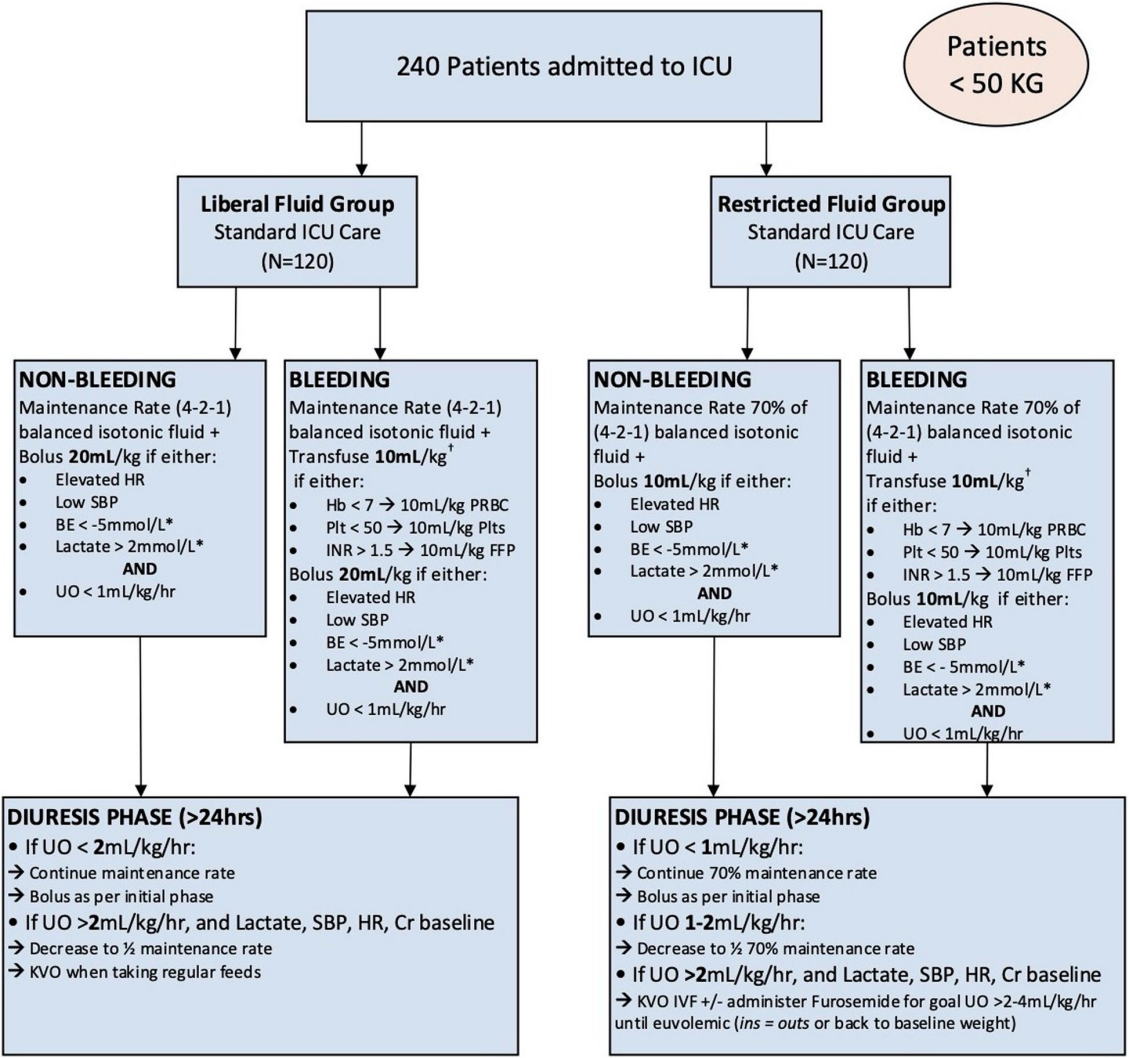
Revised treatment algorithm after first study analysis for patients < 50 kg

**Fig. 6:**
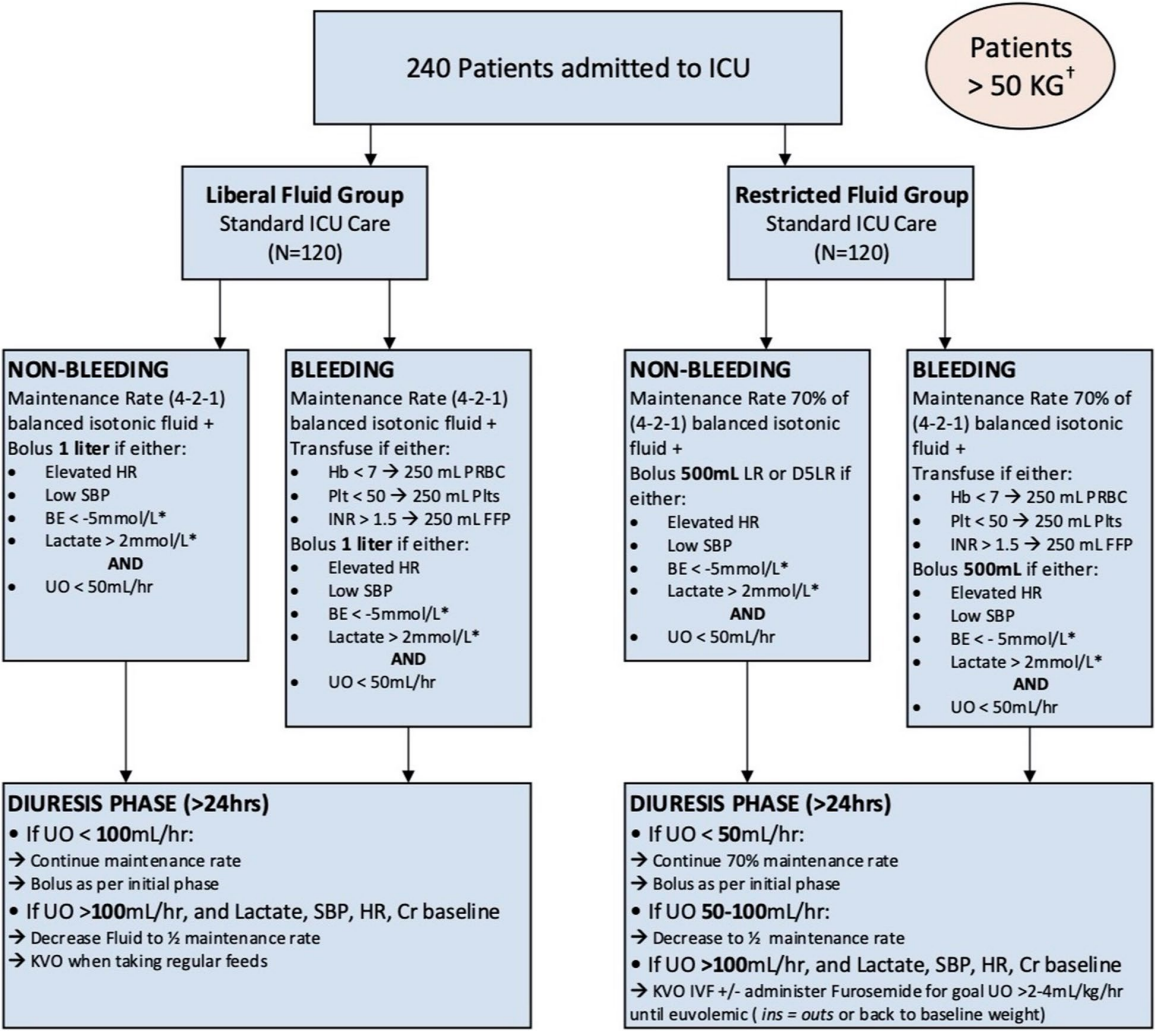
Revised treatment algorithm after first study analysis for patients > 50 kg

After data was obtained on the first 29 patients, a first study analysis was performed (Table 3). The DSMC met and concluded the study was safe and there were no study-related AEs to report. The liberal group received a median of 2.915 cc/kg/h (IQR 2.119, 4.148) of crystalloid fluid, while the restricted arm received 2.458 cc/kg/h (IQR 1.643, 3.612). This difference was deemed too negligible to lead to a treatment effect. After discussion with all co-investigators and study advisors, the volume of fluid administered to the restricted arm as designated in the algorithm was decreased to 70%, which was also more consistent with PICU practice in patients on mechanical ventilation whose fluid requirement is less, since insensible losses are reduced by the temperature-controlled humidifier connected to the ventilator. We also identified instances in which the patients showed high heart rate, a trigger for crystalloid bolus, however were not felt to be hypovolemic—they were in pain, febrile, or anxious. As such, we added oliguria as a required trigger for bolus, rather than a separate trigger, i.e., patients with tachycardia also had to have low urine output to trigger a crystalloid bolus. The treatment algorithm was changed and started being applied for the second phase of the study, from the 30th patient on (Fig. 5 depicts a revised algorithm for patients < 50 kg; Fig. 6 depicts a revised algorithm for patients < 50 kg).

**Table 3:** First study analysis–study outcomes (subjects 1–29)

Variable	Liberal (*n* = 14)	Restricted (*n* = 15)	Total (*n* = 29)	*P* value
Oxygen support	7 (50.0%)	8 (53.3%)	15(51.7%)	> 0.999
Hours on oxygen support, median (IQR)	0.700 (0.000, 2.802)	1.500 (0.000, 49.225)	1.400 (0.000, 6.500)	0.276
Mechanical ventilation	3 (21.4%)	6 (40.0%)	9 (31.0%)	0.427
Findings on chest X-ray	6 (42.9%)	9 (60.0%)	15 (51.7%)	0.466
Surgical site infection	1 (7.1%)	0 (0.0%)	1 (3.4%)	0.483
Sepsis	1 (7.1%)	2 (13.3%)	3 (10.3%)	> 0.999
Anastomotic dehiscence	2 (14.3%)	0 (0.0%)	2 (6.9%)	0.224
Days until diet resumed, median (IQR)	2.730 (1.244, 3.540)	1.750 (1.025, 4.523)	2.616 (1.164, 4.205)	0.534
Acute kidney injury	0 (0.0%)	2 (14.3%)	2 (7.4%)	0.481
Pressure ulcer	0 (0.0%)	1 (6.7%)	1 (3.4%)	> 0.999
Bleeding requiring intervention	7 (50.0%)	3 (20.0%)	10 (34.5%)	0.128
Unplanned return to the OR	3 (21.4%)	0 (0.0%)	3 (10.3%)	0.100
Other complications	2 (14.3%)	2 (14.3%)	4 (14.3%)	> 0.999
PICU LOS in hrs, median (IQR)	46.508 (39.529, 73.367)	53.000 (29.000, 130.733)	47.967 (29.933, 91.783)	0.760
Total LOS in hrs, median (IQR)	89.233 (72.821, 183.679)	118.033 (93.425, 258.967)	107.833 (75.583, 231.783)	0.275
Discharge to rehab facility	2 (14.3%)	0 (0.0%)	2 (6.9%)	0.234

### Second study analysis outcomes

In January 2021, a second study analysis of 21 patients was performed after a total of 50 patients were enrolled, and this data was analyzed. The DSMC met a second time and again found the study to be safe. No study-related adverse events were identified. The enrollment rate during this second study period was as follows: 54% (21/39, 95% CI 37–70%) of patients approached were enrolled in the study. Of the patients enrolled, 71% (15/21, 95% CI 48–89%) completed the study. This met our a priori recruitment and retention criteria for success. An analysis was performed on patients 30–50 (Table 4). The liberal group received a median volume of 2.567 cc/kg/h (IQR 1.527, 3.095) and the restricted group received 1.602 cc/kg/h (IQR 1.523, 2.434). Although not statistically significant, we observed some differences in outcomes that might be of clinical interest. Specifically, being placed on the restricted arm might have been associated with improved pulmonary outcomes—decreased need for oxygen support (50% of patients on liberal arm vs 33% of patients on the restricted arm), fewer hours on oxygen support (median 3.5 h for patients on liberal arm vs 0 h for patients on the restricted arm), and decreased need for mechanical ventilation (25% of patients on liberal arm vs 22% of patients on the restricted arm). Findings on CXR were comparable between groups with 4 patients (33%) having CXR findings in the liberal group (pneumothorax (1), hydropneumothorax (1), pleural effusion (1), atelectasis (1)) and 3 (33%) in the restricted group (pulmonary edema (1), atelectasis (2)). There was no correlation between the study arms and other complications or length of stay. Although the number of patients was small, these trends helped to refine our outcomes and set up future multicenter feasibility studies to adequately power for clinically relevant outcomes.

**Table 4:** Second study analysis–study outcomes (subjects 30–50)

Variable	Liberal, *n* = 12	Restricted, *n* = 9	Total, *n* = 21	*P* value
Oxygen support	6 (50.0%)	3 (33.3%)	9 (42.9%)	0.660
Hours on oxygen support, median (IQR)	3.500 (0.000, 9.438)	0.000 (0.000, 14.000)	0.000 (0.000, 13.000)	0.694
Mechanical ventilation	3 (25.0%)	2 (22.2%)	5 (23.8%)	> 0.999
Findings on chest X-ray	4 (33.3%)	3 (33.3%)	7 (33.3%)	> 0.999
Surgical site infection	0 (0.0%)	0 (0.0%)	0 (0.0%)	NA
Sepsis	1 (8.3%)	0 (0.0%)	1 (4.8%)	> 0.999
Anastomotic dehiscence	0 (0.0%)	0 (0.0%)	0 (0.0%)	NA
Days until diet resumed, median (IQR)	2.547 (1.683, 3.801)	2.819 (1.769, 5.028)	2.547 (1.716, 4.686)	0.509
Acute kidney injury	0 (0.0%)	0 (0.0%)	0 (0.0%)	NA
Pressure ulcer	0 (0.0%)	0 (0.0%)	0 (0.0%)	NA
Bleeding requiring intervention	1 (8.3%)	1 (11.1%)	2 (9.5%)	> 0.999
Unplanned return to the OR	0 (0.0%)	0 (0.0%)	0 (0.0%)	NA
Other complications	3 (25.0%)	4 (44.4%)	7 (33.3%)	0.397
PICU LOS in h, median (IQR)	37.933 (25.138, 76.871)	89.250 (23.233, 141.467)	43.967 (23.233, 89.250)	0.394
Total LOS in h, median (IQR)	96.992 (71.854, 147.979)	213.733 (93.367, 257.250)	113.800 (73.567, 215.183)	0.227
Discharge to rehab facility	0 (0.0%)	0 (0.0%)	0 (0.0%)	NA

After a focus meeting with the co-investigators and study advisors to discuss this second phase analysis, the algorithm was revised a second time for use in future multicenter studies. We changed the cut-off weight from 50 to 70 kg to replicate clinical practice in terms of when fluid therapy transitions from weight-based management to non-weight-based management for older children. In this revised algorithm, patients weighing 70 kg or more all received the treatment intervention based on 70 kg regardless of their weight.

Taking into account the parameters used for resuscitation in Pediatric Advanced Life Support, the trigger for intervention during the resuscitative phase—administration of crystalloid bolus—was changed from heart rate 20% above the mean for that age and systolic blood pressure 20% below the mean for that age to heart rate greater than 95th percentile or systolic blood pressure lower than 5th percentile for that age, respectively. We also decreased the urine output used to define oliguria to align it with PICU common practice. Specifically, oliguria was defined as < 1 ml/kg/h in the liberal group and < 0.5 ml/kg/h in the restricted group. Finally, we added specific limits in the diuresis phase to avoid over-diuresis, e.g., recommending diuresis only if total in/out balance of IV fluid was positive for the 24-h period.

**Fig. 7:**
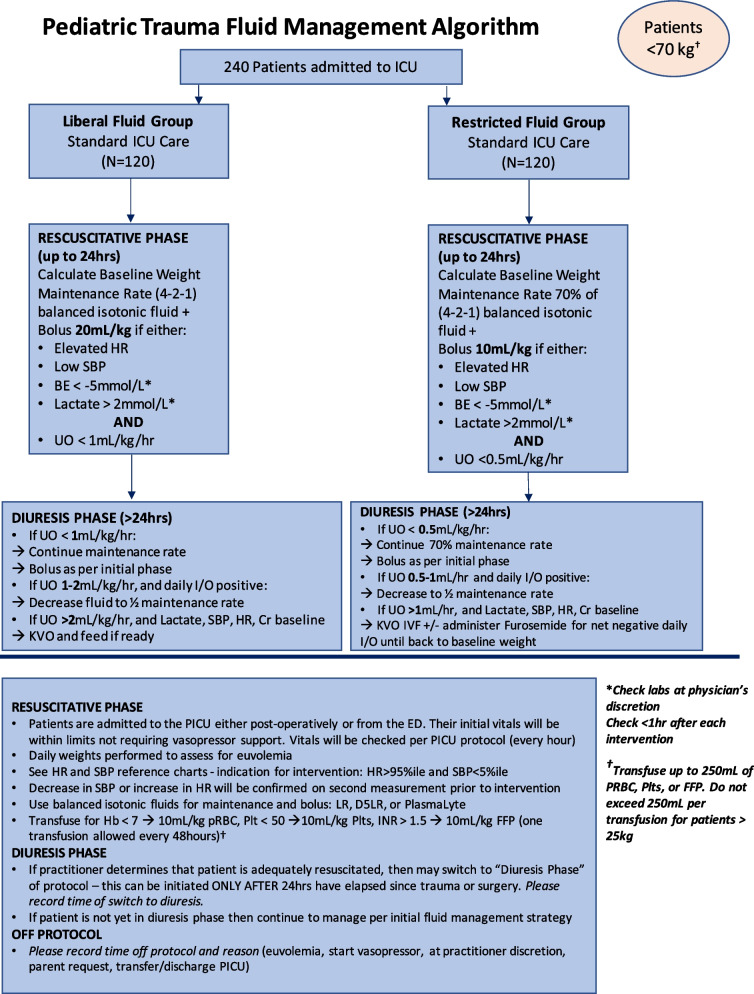
Final treatment algorithm after second analysis for patients < 70 kg

**Fig. 8:**
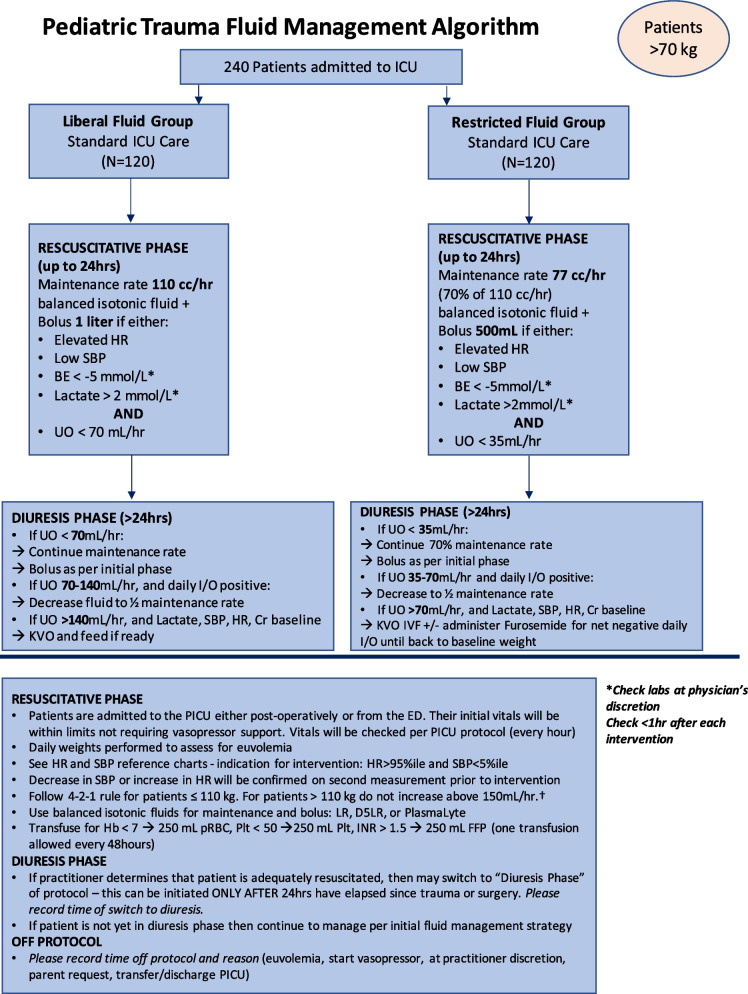
Final treatment algorithm after second analysis for patients > 70 kg

Recent data have been published providing support for bypassing crystalloid fluid altogether in favor of blood product resuscitation, and even whole-blood resuscitation in bleeding trauma patients [27, 28]. As such, we removed bleeding patients from our study. Again, in line with our goal to provide a treatment algorithm that is applicable to pediatric trauma patients in various types of settings, we determined that focusing on non-bleeding patients was appropriate since most pediatric trauma patients do not exhibit severe blood loss. Only 5–15% of pediatric trauma patients meet the criteria for massive hemorrhage, and a number of pediatric registry studies have reported a median injury severity score of 9 [28–31]. At this point, the algorithm had been vetted several times from multiple discussions with the co-investigators, and PICU and pediatric trauma advisory members. It has also been modified in response to data obtained from the pilot study and recent published studies (Fig. 7 depicts the final algorithm for patients weighing < 70 kg; Fig. 8 depicts the final algorithm for patients weighing > 70 kg) [27, 28].

## Discussion

In January 2021, we completed the analysis on a single-site pilot randomized controlled trial of 50 critically ill pediatric post-operative and trauma patients comparing high-volume (liberal arm) versus low-volume (restricted arm) fluid management strategies. We found the study to be feasible, safe, and able to produce clinically valid trends. The enrollment rate during the second study period (patients 30–50) was as follows: 54% (21/39) of patients approached were enrolled in the study. Of the patients enrolled, 71% (15/21) completed the study. This finding was consistent with our pre-study estimate of 50% willingness to participate in the study. There was buy-in from the treating physicians and adherence to the protocol in most cases. Safety was demonstrated by the lack of adverse events and renal dysfunction in patients in both analyses. An independent data safety monitoring committee confirmed the safety of the study. After changes to the algorithm were made in accordance with focus meetings with co-investigators and study advisors, the second study analysis identified a non-significant trend towards improved outcomes in the restricted fluid therapy arm, including decreased need for oxygen support, fewer hours on oxygen support, and decreased need for mechanical ventilation.

Currently, different fluid strategies are in practice because of the lack of evidence in children. Some practitioners provide fluids liberally, stating that critically ill post‐operative and trauma patients experience a systemic inflammatory response syndrome (SIRS) that causes a capillary leak of fluid out of the vascular system into surrounding tissues. These patients require large-volume fluid management to maintain intravascular volume and stay hemodynamically stable. Conversely, others use a restricted fluid strategy arguing that such high fluid volumes worsen acidosis, create local endothelial disruption, and contribute to volume overload from the leaked plasma. This leads to increased cardiac and pulmonary complications, wound complications, disturbance of the coagulation system, and delay in gastrointestinal function [32–36]. In fact, in our recent retrospective review of 200 pediatric trauma patients, a high volume of crystalloid fluids was associated with worse pulmonary and gastrointestinal outcomes as well as increased hospital length of stay [37]. Similar retrospective studies of pediatric trauma patients have also found unfavorable outcomes associated with excessive crystalloid fluid, including longer ICU and hospital length of stay, anemia, and RBC infusion requirement [38, 39]. This pilot study recognizes the lack of prospective data on fluid volume in pediatric patients following an inflammatory event (trauma or surgery) and demonstrates feasibility of a randomized controlled trial to confirm existing retrospective findings in trauma patients. While the study was not powered for nor intended to find statistically significant correlations between interventions and outcomes, we suggest that restricted crystalloid fluid management may be beneficial by identifying a trend toward improved pulmonary outcomes in the low-volume (restricted arm) fluid management group.

In 2020, the International Fluid Academy (IFA) highlighted the four recently defined phases of IV fluid therapy in critically ill patients—resuscitation, optimization, stabilization, and evacuation (R.O.S.E.). However, there is a fundamental gap in knowledge about what defines these different phases of fluid therapy in pediatric post-operative and trauma patients and how to manage their fluids during each phase of therapy. Studies are ongoing to determine the appropriate type—crystalloid, blood, other—of fluid to use for the resuscitation and optimization phases of pediatric post-operative and trauma patients. In this study, we oberserved that there are no well-defined clinical markers used by treating physicians to transition to the stabilization phase of fluid therapy. Defining specific clinical variables that correlate with the different phases of fluid therapy will be one of the next steps in our research.

### Generalizability and future applications

We consider that this study is impactful as it is the first randomized controlled study comparing restricted to liberal fluid management in critically ill pediatric post-operative and trauma patients. RCTs are challenging to perform in critically ill children. Parents are anxious about enrolling their children in research studies. Furthermore, having a child in the PICU is a stressful event for parents, making them less likely to want to participate in research studies. Yet, our enrollment rate was more than 50%. We demonstrated that the study is safe and feasible to conduct at our institution. We feel that a prospective multicenter clinical trial to determine the optimal fluid strategy in post-operative and trauma patients is urgently needed due to a lack of data in children and should be guided by the findings of this pilot study. Another strength of our study is the evidence-based nature of our treatment algorithm. It was initially designed through a rigorous review of the literature and current guidelines, with input from content experts. It was then further refined during the study. Prior to the initiation of the multicenter RCT, the fluid therapy algorithm will be verified by an expert consensus panel using the Delphi Method. The Delphi Method involves an anonymous survey of individual expert opinions with results presented to the group in a series of rounds, allowing for revisions from personal opinions and global input on the group opinion. Specifically, the following study elements will need to be taken into consideration: inclusion and exclusion criteria – whether both postoperative and trauma patients should be included in the study, what trauma patients should be excluded, what surgical patients should be included; what study interventions will be initiated in patients in the “active de-escalation” arm of the study during the stabilization and evacuation phases (e.g., use of diuretics, dose, type, length of treatment, treatment goals); and definition of complications and reliable outcomes to measure. All phases of fluid therapy must be matched with clinical, laboratory, and imaging data to determine when to de-escalate fluids in pediatric post-operative and trauma patients.

### Limitations

Although we report a trend towards improved pulmonary outcomes in the group with low-volume fluid management, the number of patients is very small, and the study was not powered to identify differences in outcomes. We plan to complete a multicenter study powered to determine the effect of fluid strategy on these outcomes. Furthermore, we adapted study interventions from the results of two study analyses and structured focus groups with co-investigators and advisors. These focus groups were composed of experts in pediatric trauma and critical care, however, they represent the expert opinions of individuals from our institution only.

A significant limitation of the study is that fluids administered prior to starting the study protocol are not considered in the total ins and outs of crystalloid fluid administered. We have not found a logistically feasible way to accurately calculate all fluids administered in the ER, OR, and PACU prior to arriving at the PICU and enrolling in the study. Therefore, this volume of fluid is not considered in the total ins and outs. Additionally, the fluids administered prior to enrollment in the study are not controlled per the protocol. We determined that significant variation in fluid strategy would confound the comparison of fluid volumes administered to the two groups of patients; however, analysis including pre-study fluids should be undertaken. Our institution has obtained a new electronic medical record since the end of the feasibility study that will facilitate the collection of fluid data from different divisions. We will be collecting this data in the planned multicenter study.

Another limitation of the study involves our enrollment logs. Due to incomplete documentation, enrollment logs for patients from the first study period (patients 1–29) were not able to be included in the study. We have accurate enrollment data for patients in the second study period (patients 30–50). These have been included in the study and are encouraging as we were able to enroll more than half (54%) of patients approached for the study.

The total time spent on protocol was limited. Over the course of the two studies the average time patients spent on the study was a median of 22.800 h for the liberal group and 26.5300 h for the restricted group. The pilot study, which was intended to demonstrate feasibility and safety, was limited to 50 patients. A minimum time on the study was not required for inclusion. For the multicenter study, we will enroll patients who are anticipated to be admitted to the PICU for more than 24 h to allow there to be an effect of the intervention.

## Conclusion

Crystalloid fluid therapy is a cornerstone of the management of critically ill pediatric post-operative and trauma patients. No guidelines exist to guide fluid management in these patients. Prospective studies are needed. We have demonstrated the feasibility and safety of conducting an RCT comparing restricted to liberal fluid management in these patients at our institution. In response to our study analyses, we have adapted our fluid algorithm to make it more applicable to current clinical practice with a greater chance of protocol adherence and study completion. We plan to use the results of the study to design a multicenter RCT that will provide substantial data and guidance in fluid management of pediatric post-surgical and trauma and improve outcomes.

### Supplementary Information

**Additional file 1.** Supplementary materials.

## Data Availability

The datasets used and/or analyzed during the current study are available from the corresponding author on reasonable request.
