# Supplementary material for: Pilot randomized controlled trial of restricted versus liberal crystalloid fluid management in pediatric post-operative and trauma patients
Source: Pilot Feasibility Stud. 2023 Nov 8;9:185. doi: 10.1186/s40814-023-01408-w (PMC10631167; doi:10.1186/s40814-023-01408-w)
Supplement: Supplementary file 1 — Additional file 1. Supplementary materials. [file 40814_2023_1408_MOESM1_ESM.docx]

**Supplementary materials**

**Full list of variables recorded in REDCap:**

Demographics: age, bleeding status, gender, weight, height, diagnosis, and comorbidities.

Surgical specialty was recorded for surgical patients.

Injury Severity Score (ISS), Glascow Coma Scale (GCS) on presentation, mechanism of injury, region of injury, and type of injury (blunt or penetrating) was recorded for trauma patients.

Clinical variables: fluid intake (crystalloid and colloid fluid), output (urine, stool, GI and drain), requirement for and length of oxygen support, requirement for and length of mechanical ventilation, chest X-Ray findings, pupillary reaction, maximum heart rate, minimum systolic blood pressure, maximum creatinine, abnormal laboratory values, time until diet resumed, PICU and hospital length of stay, and PICU discharge disposition.

Complications while on the protocol: pneumonia, ventilator-associated pneumonia, ARDS, bloodstream infection, central line associated bloodstream infection, surgical site infection, sepsis, anastomotic dehiscence, abdominal compartment syndrome, extremity compartment syndrome, deep vein thrombosis, pulmonary embolism, acute kidney injury, pressure ulcer, catheter associated urinary tract infection, bleeding requiring intervention, unplanned return to the operating room, other complications and death.

**Variables analyzed by our biostatistician and included in the results tables:**

Demographics: age, bleeding status, gender, weight, height, and comorbidities.

Surgical specialty was recorded for surgical patients.

Clinical variables: fluid intake (crystalloid fluid), requirement for and length of oxygen support, requirement for mechanical ventilation, chest X-Ray findings**,** time until diet resumed**,** PICU and hospital length of stay, and PICU discharge disposition.

Complications while on the protocol: surgical site infection, sepsis, anastomotic dehiscence, acute kidney injury, pressure ulcer, bleeding requiring intervention, unplanned return to the operating room, other complications.
